# Adaptor Protein 1A Facilitates Dengue Virus Replication

**DOI:** 10.1371/journal.pone.0130065

**Published:** 2015-06-19

**Authors:** Umpa Yasamut, Nopprarat Tongmuang, Pa-thai Yenchitsomanus, Mutita Junking, Sansanee Noisakran, Chunya Puttikhunt, Justin Jang Hann Chu, Thawornchai Limjindaporn

**Affiliations:** 1 Division of Molecular Medicine, Department of Research and Development, Faculty of Medicine Siriraj Hospital, Mahidol University, Bangkok, Thailand; 2 Graduate Program in Immunology, Department of Immunology, Faculty of Medicine Siriraj Hospital, Mahidol University, Bangkok, Thailand; 3 Medical Biotechnology Unit, National Center for Genetic Engineering and Biotechnology, National Science and Technology Development Agency, Bangkok, Thailand; 4 Laboratory of Molecular RNA Virology and Antiviral Strategies, Department of Microbiology, Yong Loo Lin School of Medicine, National University Health System, National University of Singapore, Singapore; 5 Department of Anatomy, Faculty of Medicine Siriraj Hospital, Mahidol University, Bangkok, Thailand; University of Rochester, UNITED STATES

## Abstract

Rearrangement of membrane structure induced by dengue virus (DENV) is essential for replication, and requires host cellular machinery. Adaptor protein complex (AP)-1 is a host component, which can be recruited to components required for membrane rearrangement. Therefore, dysfunction of AP-1 may affect membrane organization, thereby decreasing replication of virus in infected cells. In the present study, AP-1-dependent traffic inhibitor inhibited DENV protein expression and virion production. We further clarified the role of AP-1A in the life cycle of DENV by RNA interference. AP-1A was not involved in DENV entry into cells. However, it facilitated DENV RNA replication. Viral RNA level was reduced significantly in Huh7 cells transfected with AP-1A small interfering RNA (siRNA) compared with control siRNA. Transfection of naked DENV viral RNA into Huh7 cells transfected with AP-1A siRNA resulted in less viral RNA and virion production than transfection into Huh7 cells transfected with control siRNA. Huh7 cells transfected with AP-1A siRNA showed greater modification of membrane structures and fewer vesicular packets compared with cells transfected with control siRNA. Therefore, AP-1A may partly control DENV-induced rearrangement of membrane structures required for viral replication.

## Introduction

Dengue virus (DENV) is a positive-stranded RNA virus in the *Flaviviridae* family, which is transmitted by mosquito vectors. The genome of DENV has sequences encoding structural proteins including capsid (C), pre-membrane protein (prM), and envelope (E), and non-structural proteins (NS) including NS1, NS2A, NS2B, NS3, NS4A, NS4B and NS5 [1]. DENV consists of four serotypes, and secondary infection by different serotypes of DENV contributes to severe dengue [2]. Patients with dengue hemorrhagic fever often present with plasma leakage, hemoconcentration, thrombocytopenia, and hemorrhagic tendencies. Additionally, serious complications of dengue hemorrhagic fever, such as organ failure, may lead to dengue shock syndrome [1–3]. Currently, there are no effective vaccines or antiviral drugs available; therefore, a better understanding of dengue pathogenesis is required.

DENV needs host cellular machinery for its replication. It binds to receptors and enters host cells by clathrin-mediated endocytosis [4–16]. Reduced pH in the endosomes induces fusion of viral and host cell membranes, thereby releasing DENV RNA into the cytoplasm [17]. Viral replication occurs on the network of modified endoplasmic reticulum (ER) membranes, including vesicular packets, virus-induced vesicles, and convoluted membranes [18–20]. Immature viral particles are transported through the trans-Golgi network (TGN) and mature virions are generated after cleavage of prM protein by host furin. Mature viruses are finally released from the host cells by exocytosis [21].

Host genes are important for the viral life cycle, including endocytosis, virus-induced membrane rearrangement, viral RNA replication and translation, and virion assembly and production. RNA interference (RNAi) is commonly used as a tool to identify the role of host proteins during DENV infection [4, 20, 22–28]. One of the host protein complexes identified is adaptor protein complex [4, 22, 24].

Adaptor protein complex (AP) was originally identified as a component of the clathrin-coated vesicles in the brain [29, 30]. Each member of AP has two large subunits (γ/β1, α/β2, δ/β3, ε/β4 or ζ/β5), one medium subunit (μ1–β5), and one small subunit (σ1–σ5). AP-1A consists of one medium subunit (μ1A), two large subunits (β1 and γ), and one small subunit (σ1). AP-1B consists of one medium subunit (μ1B), two large subunits (β1 and δ), and one small subunit (σ1). The μ subunit mediates a selection of cargo proteins via its binding with tyrosine-based sorting motif on the cargo protein [31–33]. AP-1A is expressed ubiquitously and regulates the TGN-basolateral plasma membrane transport. AP-1B is expressed in epithelial cells and regulates the basolateral transport of proteins from the recycling endosomes [34–36]. AP-1A can be recruited to components required for membrane rearrangement. In addition, interactions between AP-1A and viral proteins are reported [37, 38]. Therefore, dysfunction of AP-1A may affect membrane organization, thereby decreasing viral replication in DENV-infected cells.

## Materials and Methods

### Cell lines, virus, and antibodies

Human hepatocellular carcinoma (Huh7) cells were obtained from the JCRB Cell Bank (Osaka, Japan) and cultured in RPMI 1640 (Gibco, Carlsbad, CA, USA) supplemented with 10% heat-inactivated fetal bovine serum (FBS; Gibco), 1% non-essential amino acid (Gibco), 37 μg/ml penicillin (Sigma, St Louis, MO, USA) and 60 μg/ml streptomycin (Sigma) at 37°C in a 5% CO_2_ incubator with a humidified atmosphere. Human lung carcinoma (A549) cells were obtained from ATCC and cultured in DMEM (Gibco, Carlsbad, CA, USA) supplemented with 10% heat-inactivated fetal bovine serum (FBS; Gibco), 1% non-essential amino acid (Gibco), 1mM sodium pyruvate (Gibco), 37 μg/ml penicillin (Sigma, St Louis, MO, USA) and 60 μg/ml streptomycin (Sigma) at 37°C in a 5% CO_2_ incubator with a humidified atmosphere. Propagation of DENV-1 (Hawaii), DENV-2 strain 16681, DENV-3 (H87), and DENV-4 (H241) was performed in C6/36 mosquito cells (ATCC). DENV-2 was used in all experiments. Mouse monoclonal antibodies specific for DENV E (clones 3H5 and 4G2), DENV prM (clone 1C3), and DENV NS1 (clone NS1-3F.1) were produced from previously established hybridoma cells [39–41]. Mouse polyclonal antibody specific for AP-1A (μ1A subunit) was purchased from Abnova (Taipei, Taiwan). Mouse monoclonal antibody specific for β-actin and goat polyclonal antibody specific for GRP78 were purchased from Santa Cruz Biotechnology (Santa Cruz, CA, USA).

### AP-1-dependent traffic inhibitor and DENV infection

Huh7 cells or A549 cells were infected with DENV-2 at a multiplicity of infection (MOI) of 1 for 2 h. Excess viruses were removed and cells were washed three times with PBS. DENV-infected or mock-infected cells were incubated with AP-1-dependent traffic inhibitor (A5) (Merck KGaA, Darmstadt, Germany) [42] at a final concentration of 0, 25, 50, 100 or 200 μM in 2% FBS–RPMI 1640 for 24 h. DENV-infected Huh7 cells were harvested for measuring viral protein expression by western blotting using antibody to DENV E (4G2). The culture supernatants were also collected to measure the amount of DENV production by a focus forming unit (FFU) assay, as described previously [43]. Cell viability was determined by PrestoBlue cell viability assay (Invitrogen, Carlsbad, CA, USA). To test the effect of A5 to ER stress pathway, DENV-infected or mock-infected cells were incubated with A5 at a final concentration of 0, 100 or 200 μM in 2% FBS–RPMI 1640 for 48 h. Clear lysates were subjected to western blot analysis using antibody to GRP78, which is a maker for ER stress (S1 Fig). To establish whether AP-1-mediated traffic was involved in production of four serotypes of DENV, Huh7 cells were infected with DENV-1, -2, -3 or -4 at a MOI of 1 for 2 h. Unbound virus was removed by washing with PBS. DENV-infected Huh7 cells were incubated with A5 (200 μM) or culture medium (control) for 24 h. Virus titer in culture supernatants was measured by FFU assay [43].

### Knockdown of AP-1A

Huh7 cells were seeded onto a 24-well plate in culture medium without antibiotics at a concentration of 8×10^4^ cells/well. Twenty-four hours later, the medium was replaced with fresh RPMI 1640 medium and the cells were transfected with duplex AP-1A-specific siRNA (AP1M1 (μ1A) siRNA: 5-CCGAAGGCAUCAAGUAUCGGAAGAA-3, Invitrogen) or control siRNA (Cat.No. 12935–300; Invitrogen) using LipofectamineRNAi Max (Invitrogen). After incubation with siRNA (100 nM) for 6 h, the cells were supplemented with maintenance medium and incubated for a further 24 h. The second round of siRNA transfection was performed. mRNA and protein expression of AP-1A and β-actin was subsequently verified by real-time reverse transcription polymerase chain reaction (RT-PCR) (Lightcycler RNA Amplification Kit; Roche, Basel, Switzerland) and western blot analysis [44] using AP-1A and β-actin antibodies, respectively. Cell viability was measured by trypan blue exclusion, as described previously [45].

### Binding assay

Huh7 cells transfected with AP-1A siRNA or control siRNA were detached by trypsinization. After washing three times with PBS, cells were blocked with 1% bovine serum albumin (BSA)–PBS for 30 min and incubated with DENV-2 at a MOI of 1 or MOI of 10 for 30 min on ice to prevent endocytosis. Cells were washed twice with 1% BSA–PBS and incubated with anti-DENV E antibody (3H5) for 30 min on ice. After washing, rabbit anti-mouse IgG conjugated with fluorescein isothiocyanate (dilution 1:50) was added and incubated for 30 min on ice. Cells were washed three times with 1% BSA–PBS and resuspended in 350 μl of 1% paraformaldehyde–PBS. Virus binding was finally counted by the mean fluorescence intensity of surface DENV-E-positive cells analyzed by flow cytometry.

### Internalization assay

Huh7 cells transfected with AP-1A siRNA or control siRNA were incubated with DENV-2 at a MOI of 1 or MOI of 10 for 2 h at 37°C to allow penetration of DENV. To remove excess virus, cells were washed three times with PBS. RNA was extracted from DENV-infected Huh7 cells using the High Pure RNA isolation kit (Roche) and 0.5 μg RNA was reverse transcribed by random hexamer primers from the Superscript III cDNA synthesis kit (Invitrogen). cDNA was amplified by PCR using SYBR Green I Master Mix and primers specific to DENV E. Real-time RT-PCR was performed by LightCycler 480 II (Roche) with: (i) 40 amplification cycles of denaturation at 95°C for 10 s, annealing at 60°C for 10 s, and extension at 72°C for 10 s; and (ii) melting curve and cooling steps as recommended by the manufacturer. Relative levels of human AP-1A mRNA and viral RNA expression were determined by normalization to the expression levels of human β-actin according to the 2^–ΔΔCt^ method [46].

### Real-time RT-PCR

RNA was extracted from DENV-infected Huh7 cells, which were transfected with AP-1A-specific siRNA or control siRNA using the High Pure RNA isolation kit (Roche). Reverse transcription was performed using total RNA (1 μg) and SuperScript III reverse transcriptase (Invitrogen). Oligo(dT) 20 primer or random hexamer primers were used for synthesis of cDNA template for determination of human AP-1A, AP-2, AP-3A and β-actin mRNA, as well as DENV RNA. Real-time RT-PCR was performed using primers (S1 Table) by LightCycler 480 II (Roche) with: (i) 40 amplification cycles of denaturation at 95°C for 10 s, annealing at 60°C for 10 s, and extension at 72°C for 10 s; and (ii) melting curve and cooling steps as recommended by the manufacturer’s instructions. Relative levels of human AP-1A, AP-2, AP-3A mRNA and viral RNA expression were determined by normalization to the expression levels of human β-actin according to the 2^–ΔΔCt^ method [46].

### Viral RNA transfection

DENV RNA was isolated from culture supernatant of DENV-infected C6/36 cells using the High Pure RNA isolation kit (Roche). In a 24-well plate, cells transfected with AP-1A siRNA or control siRNA were transfected with DENV RNA (0.5 μg) using Lipofectamine 2000. At 4 h post-transfection, transfection reagent was removed and replaced with complete RPMI 1640 medium. Cells were harvested at 6, 12 and 24 h post-transfection for detection of viral RNA by real-time RT-PCR. The culture supernatant was collected at 24 h and 48 h post-transfection for FFU assay [43].

### Indirect immunofluorescence staining

Huh7 cells were plated on coverslips, transfected with a plasmid containing AP-1A [47]and infected with DENV for 24 h. The cells were fixed with 4% paraformaldehyde-PBS (Sigma-Aldrich) and 0.2% Triton X-100-PBS (Sigma) for 10 min at room temperature. The cells were incubated with mouse monoclonal anti-double-stranded RNA (anti-dsRNA) antibody (English & Scientific Consulting), rabbit polyclonal anti-GFP antibody (Abcam) for 1 h at room temperature. Upon removal of primary antibodies, cells were incubated with secondary antibodies (Alexa Fluor 488-conjugated donkey anti-rabbit IgG (Invitrogen), Alexa Fluor 594-conjugated donkey anti-mouse IgG (Invitrogen) for 30 min at room temperature. Hoechst 33342 (Molecular Probe) was used to stain cell nuclei. The stained cells were visualized by a confocal laser-scanning microscope (LSM 510 Meta).

### Translation assay

pRL-SV40 vector (Promega), which contains *Renilla* luciferase gene, was linearized with *Xba*I. One μg of purified DNA was subjected to *in vitro* transcription using the RiboMAX Large Scale RNA Production System-T7 (Promega) in the presence of 20 mM m^7^G(5′)ppp(5′)G RNA cap structure analog (New England BioLabs, Ipswich, MA, USA) and resultant RNA product was purified using RNeasy Mini Kit (QIAGEN, Hilden, Germany). To determine the effect of AP-1A knockdown on translation, Huh7 cells were transfected twice with AP-1A-specific siRNA or control siRNA in a 96-well plate within a 24-h interval using Lipofectamine 2000 (Invitrogen) according to the manufacturer’s instructions. After the second round of siRNA transfection, cells were transfected with 2.5 nM reporter RNA using Lipofectamine 2000 (Invitrogen) followed by replacement with fresh culture medium at 4 h later. Following 8 h after transfection with reporter RNA, cells were harvested and determined for *Renilla* luciferase expression using Luciferase Reporter Assay System (Promega). *Renilla* luciferase signal was measured by Synergy H1 Hybrid multi-mode microplate reader (BioTek, Winooski, VT, USA).

### Western blotting

Clear lysates prepared from Huh7 cells transfected with AP-1A siRNA or control siRNA were mixed with 4× loading buffer [50 mMTris–HCl (pH 6.8), 2% SDS, 0.1% bromophenol blue and 10% glycerol] and heated at 95°C for 5 min. Proteins in the samples were subjected to 10% SDS-PAGE and transferred to nitrocellulose membranes (GE Healthcare Life Sciences,Freiburg, Germany) as described previously [28]. The membranes were incubated with 5% skimmed milk in PBS or in Tris-buffered saline with 0.1% Tween 20 (TBST) for 1 h to block non-specific binding and with mouse monoclonal antibodies specific for DENV E (clones 4G2), DENV PrM (clone 1C3), and DENV NS1 (clone NS1-3F.1) or human β-actin at 4°C overnight. The membranes were washed three times with PBS or TBST and incubated with horseradish peroxidase (HRP)-conjugated rabbit anti-mouse immunoglobulin antibody (DAKO, Santa Clara, CA, USA) at a dilution of 1:1000 for 1 h at room temperature, followed by three further washes. Proteins were visualized using an enhanced chemiluminescence detection kit (SuperSignal West Pico Chemiluminescent Substrate; Thermo Scientific, Waltham, MA, USA). Relative expression levels of human AP-1A, and DENV proteins were assessed by normalization of their protein band intensities to human β-actin intensity using ImageJ software (National Institutes of Health, Bethesda, MD, USA).

### Transmission electron microscopy (TEM)

Huh7 cells transfected with AP-1A siRNA or control siRNA were infected with DENV-2 at a MOI of 10 for 2 h. Cells were washed three times with PBS and incubated in maintenance medium (2% FBS–RPMI 1640) for 24 h. Cells were fixed with 2% paraformaldehyde/2% glutaraldehyde in PBS for 24 h at 4°C, post-fixed with 1% osmium tetroxide and 1% potassium ferrocyanide for 1 h at room temperature, followed by dehydration in a graded series of ethanol (25%, 50%, 75%, 95% and 100%) for 20 min. Cells were embedded in LRW resin and incubated at 60°C for polymerization for 48 h. Sections were obtained with a Reichert–Jung Ultracut E Ultramicrotome and diamond knife, counterstained with uranyl acetate and lead citrate for 10 min each, and examined with a JEOL-1010 transmission electron microscope (JEOL USA, Peabody, MA, USA).

### Lipid complementation assay

Huh7 cells were infected with DENV-2 at MOI of 1 for 2 h. Excess viruses were removed and cells were washed three times with PBS. DENV-infected were incubated with A5 (200 μM) in the presence of oleic acid-BSA (Sigma) or fatty acid free-BSA (Sigma) for 24 h. The culture supernatants were collected to measure the amount of DENV production by a focus forming unit (FFU) assay, as described previously [43].

### FFU assay

Supernatants collected from DENV-infected Huh7 cells transfected with AP-1A siRNA or control siRNA were assessed for DENV production. Vero cells were seeded onto a 96-well plate (Sigma) at 3×10^4^ cells/well in minimal essential medium (MEM) supplemented with 10% FBS, 2 mM l-glutamine, 36 μg/ml penicillin and 60 μg/ml streptomycin, and cultured at 37°C in a 5% CO_2_ incubator for 24 h. The medium was removed from each well. DENV was serially diluted 10-fold in MEM containing 3% FBS, 2 mMl-glutamine, 36 μg/ml penicillin and 60 μg/ml streptomycin, added to each well (100 μl/well), and incubated at 37°C in a 5% CO_2_ incubator for 2 h. Overlay medium (MEM containing 3% FBS, 2 mMl-glutamine, 2% carboxy methyl cellulose, 10% tryptose phosphate broth, 37 μg/ml penicillin and 60 μg/ml streptomycin was added to each well (100 μl/well), and the culture was incubated for 3 days. The medium was discarded from DENV-infected cells. The adherent cells were washed three times with PBS (pH 7.4), fixed with 3.7% formaldehyde (BDH Laboratory Supplies, Poole, UK) in PBS at room temperature for 10 min, followed by an additional 10 min permeabilization with 1% Triton X-100 (Fluka, Steinheim, Switzerland). The cells were incubated sequentially with mouse anti-DENV E monoclonal antibody (clone 4G2) at 37°C for 1 h and HRP-conjugated rabbit anti-mouse immunoglobulins (DAKO) at a dilution of 1:1000 in PBS containing 2% FBS and 0.05% Tween-20 in the dark at 37°C for 30 min. To develop an enzymatic reaction, the cells were incubated with a substrate solution containing 0.6 mg/ml diaminobenzidine, 0.03% H_2_O_2_ and 0.08% NiCl_2_ in PBS at room temperature in the dark for 5 min. After washing three times with PBS, dark brown foci of the DENV-infected cells were counted under a light microscope. Virus titers were reported as FFU/ml.

### Statistical analysis

Data were statistically analyzed by unpaired *t* test, with the use of GraphPad Prism version 5.0 (San Diego, CA, USA). Results were expressed as mean and standard error of the mean (SEM) and P<0.05 was considered significant.

## Results

### Inhibition of DENV production by AP-1-dependent traffic inhibitor

**Fig 1:**
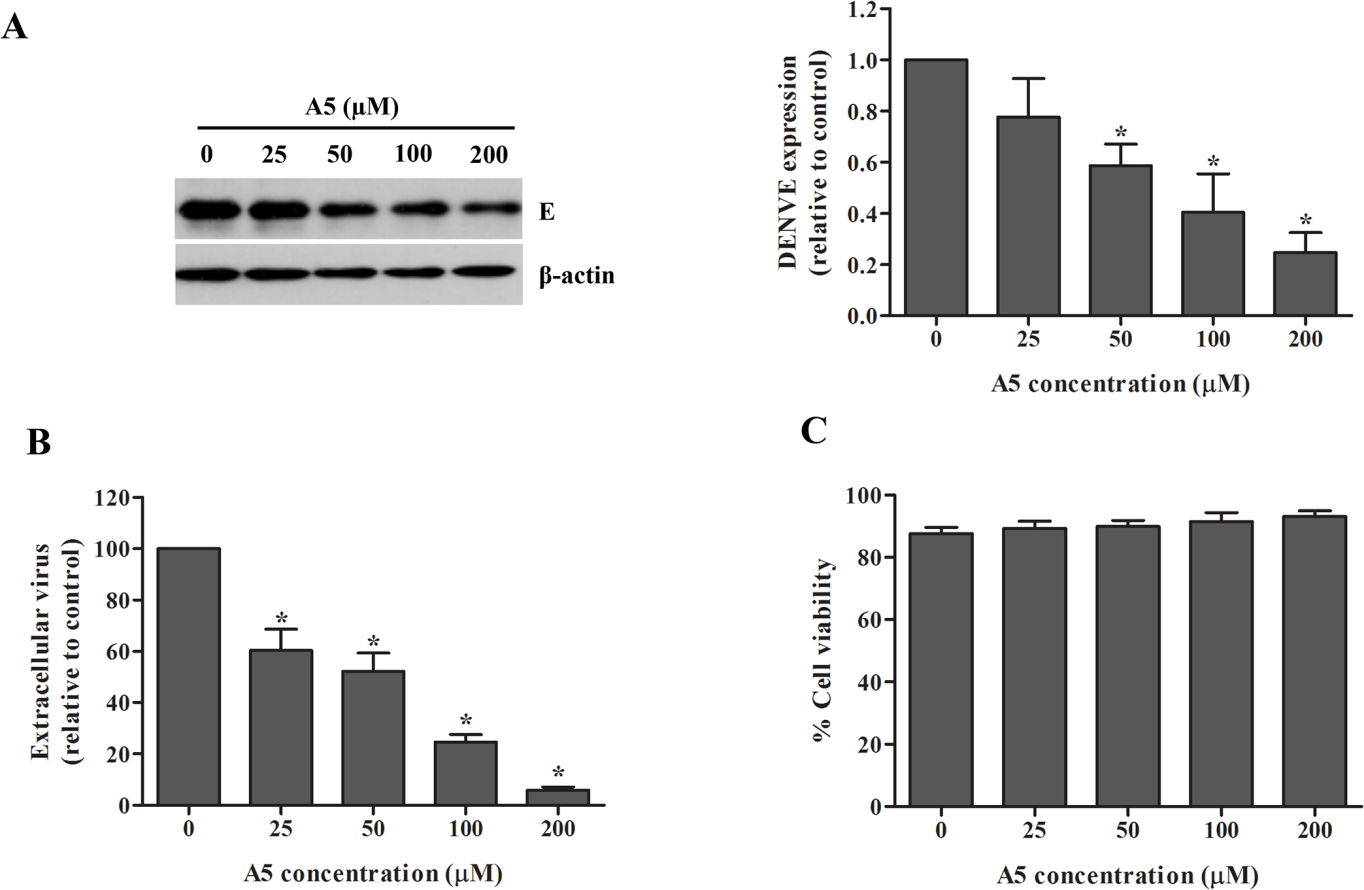
AP-1-dependent traffic inhibitor, A5, inhibited DENV production in Huh7 cells. Huh7 cells were infected with DENV-2 at a MOI of 1 for 2 h. Unbound virus was removed by washing with PBS. Mock- or DENV-infected cells were incubated with A5 at 0, 25, 50, 100 and 200 μM for 24 h. (A) DENV envelope protein in cell lysates was examined at 24 h post-treatment with A5 by western blotting. Band intensity of DENV envelope protein was quantified using Image J software. (B) Virus titer in culture supernatants was measured by FFU assay. (C) Viability of DENV-infected cells was measured using PrestoBlue cell viability reagent. Statistical significance was analyzed using the unpaired *t* test. *P<0.05. Error bars represent SEM from three independent experiments.

**Fig 2:**
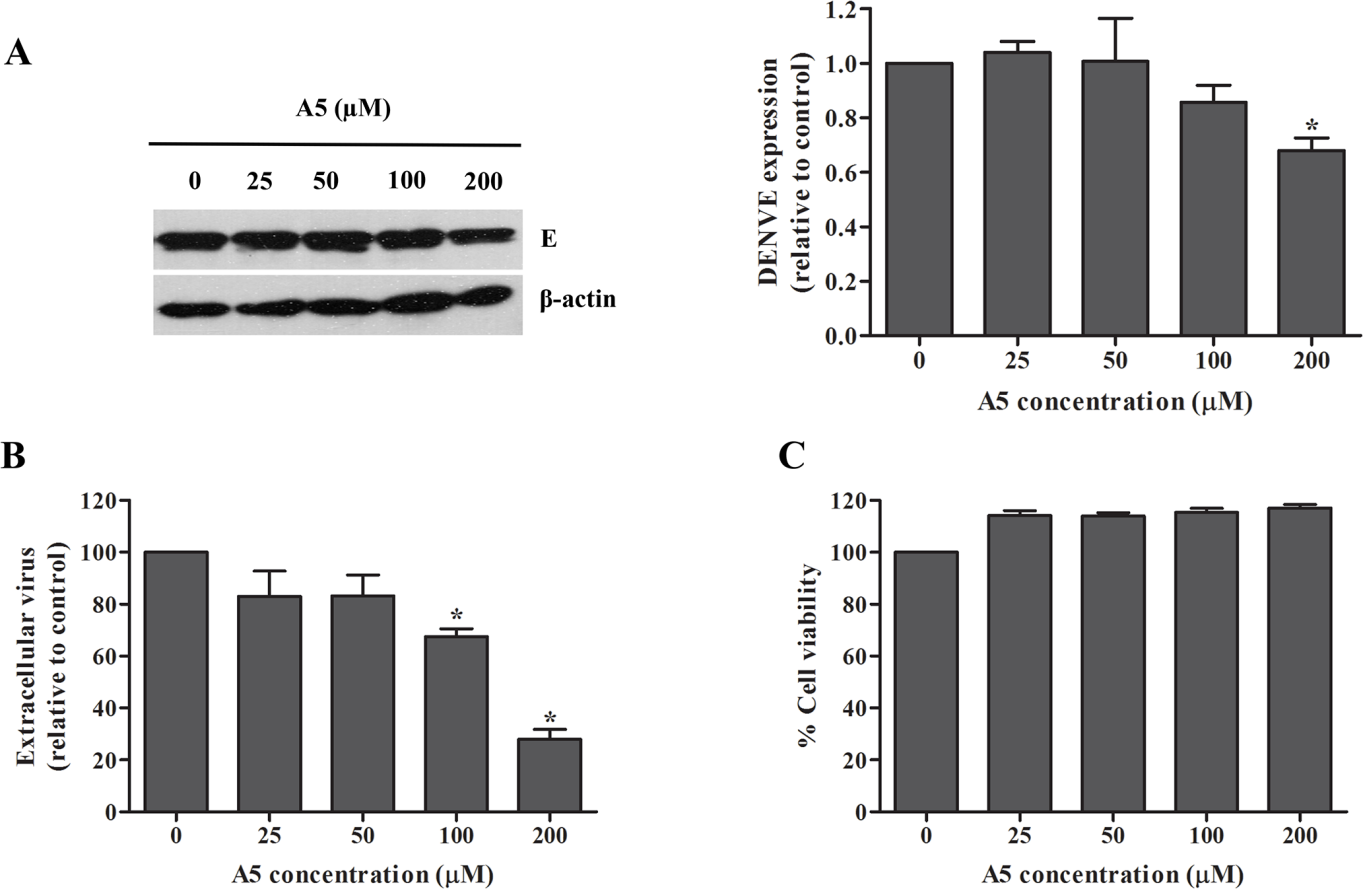
AP-1-dependent traffic inhibitor, A5, inhibited DENV production in A549 cells. A549 cells were infected with DENV-2 at a MOI of 1 for 2 h. Unbound virus was removed by washing with PBS. Mock- or DENV-infected cells were incubated with A5 at 0, 25, 50, 100 and 200 μM for 24 h. (A) DENV envelope protein in cell lysates was examined at 24 h post-treatment with A5 by western blotting. Band intensity of DENV envelope protein was quantified using Image J software. (B) Virus titer in culture supernatants was measured by FFU assay. (C) Viability of DENV-infected cells was measured using PrestoBlue cell viability reagent. Statistical significance was analyzed using the unpaired *t* test. *P<0.05. Error bars represent SEM from three independent experiments.

AP-1-dependent traffic inhibitor (A5) was previously shown to inhibit transport between TGN and Golgi in yeast [42]. We first determined whether A5 can inhibit DENV infection. DENV-infected Huh7 cells were incubated with different concentrations of A5. DENV viral protein synthesis was determined by western blotting and DENV production was measured by FFU assay. Although the viability of the cells was comparable (Fig 1C), A5 inhibited DENV protein synthesis (Fig 1A) and DENV production (Fig 1B) in a dose-dependent manner. Whether this effect was occurred in different cell line, DENV-infected A549 cells were tested in the presence or absence of A5. The result shows that A5 inhibited DENV protein synthesis (Fig 2A) and DENV production (Fig 2B) without affecting cell viability (Fig 2C). Data suggest that AP-1 is involved in DENV protein synthesis and production of dengue infectious virions.

### AP-1A is not involved in steps of binding and internalization

**Fig 3:**
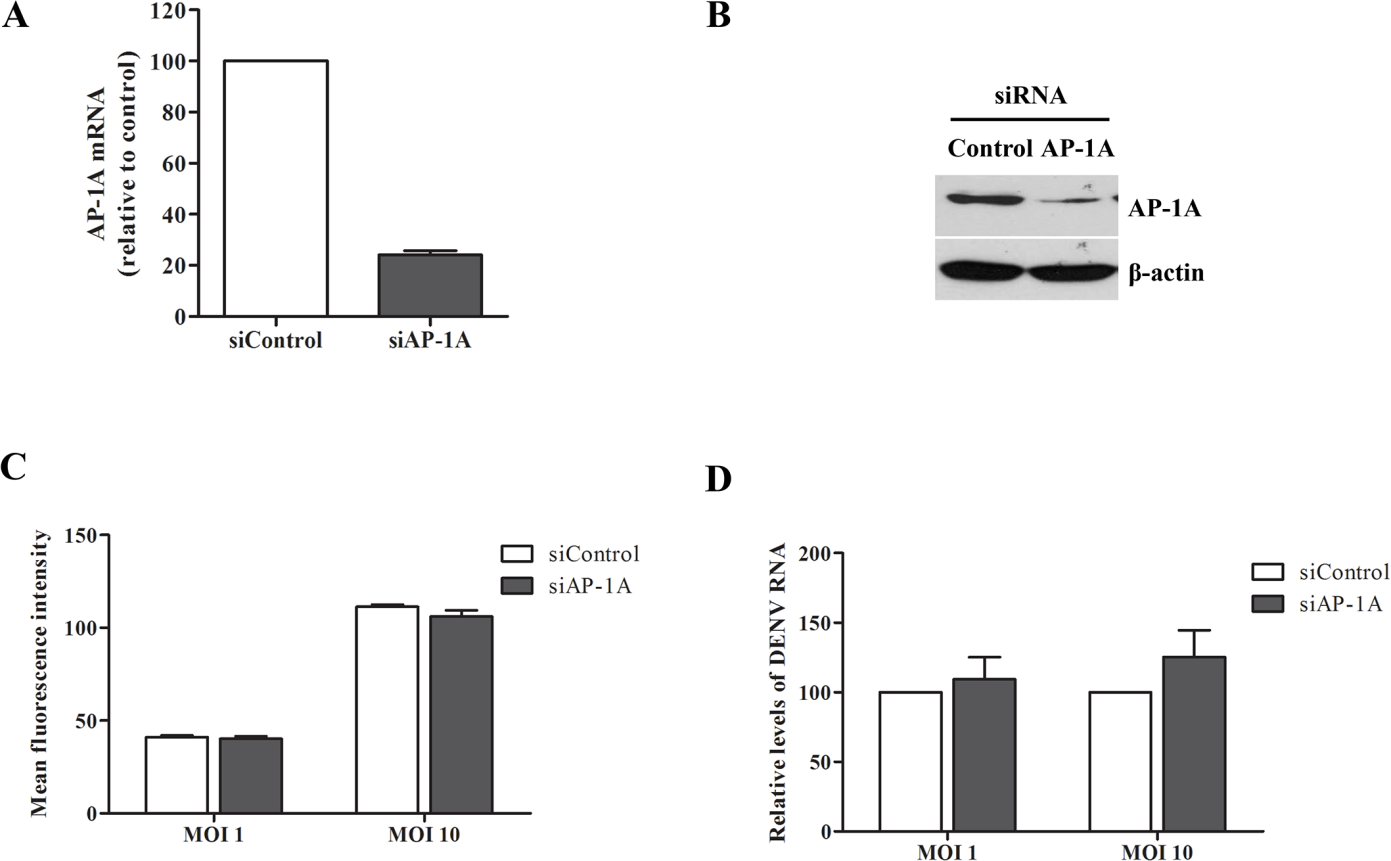
AP-1A was not involved in DENV binding and internalization. (A) Knockdown efficiency of AP-1A siRNA in Huh7 cells was examined by real-time RT-PCR at 48 h after second transfection. (B) AP-1A protein was measured by western blotting. (C) Quantification of DENV binding on Huh7 cells transfected with AP-1A siRNA. Cells transfected with control siRNA and AP-1A siRNA were incubated with DENV-2 at a MOI of 1 for 30 min on ice. Cells were surface stained with antibody to DENV E, followed by staining with the rabbit anti-mouse IgG conjugated with fluorescein isothiocyanate. The surface E-positive cells were analyzed by flow cytometry. (D) Viral internalization was determined by detecting DENV RNA at 2 h post-infection using real-time RT-PCR. Statistical significance was analyzed using unpaired *t* test (*P<0.05). Error bars represent SEM from three independent experiments.

For the AP-1A knockdown experiment, Huh7 cells were transfected twice with duplex AP-1A-specific siRNA or control siRNA for 24 h. After the second transfection, expression of AP-1A and β-actin mRNA was measured by real-time RT-PCR. Expression of AP-1A and β-actin protein was determined by western blotting using anti-AP-1A and anti-β-actin, respectively. Cell viability was determined by trypan blue exclusion assay. AP-1A mRNA expression and protein expression were reduced in Huh7 cells transfected with AP-1A siRNA compared with control siRNA (Fig 3A and 3B).

For the binding assay, Huh7 cells transfected with AP-1A siRNA or control siRNA were incubated with DENV at a MOI of 1 or at a MOI of 10 at 4°C to prevent viral internalization. Viral binding was determined by DENV E surface staining and analyzed by flow cytometry. The intensity of DENV-E-positive cells in Huh7 cells transfected with AP-1A siRNA or control siRNA was similar (Fig 3C). Therefore, AP-1A may not be involved in the binding of DENV E to Huh7 cells.

For the internalization assay, Huh7 cells transfected with AP-1A siRNA or control siRNA were incubated with DENV at a MOI of 1 or at a MOI of 10 at 37°C for 2 h to allow endocytosis. Viral RNA was determined by real-time RT-PCR. The results indicated that the viral RNA in AP-1A siRNA-transfected Huh7 cells or in control siRNA-transfected Huh7 cells was not significantly altered (Fig 3D). Therefore, AP-1A may not be involved in the internalization of DENV into Huh7 cells.

### AP-1A facilitates DENV RNA replication

**Fig 4:**
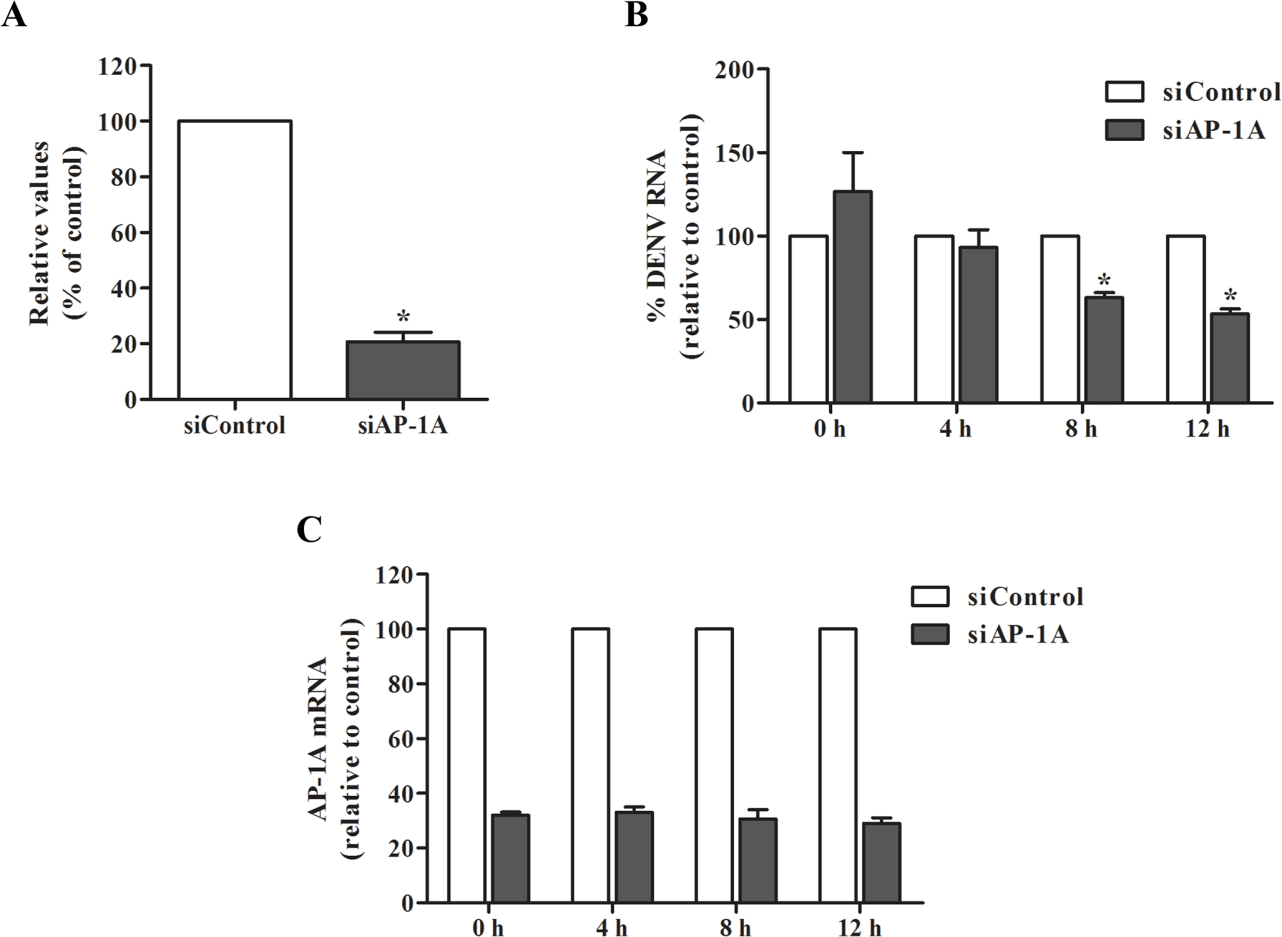
Silencing of AP-1A reduced DENV RNA level. (A) DENV RNA level was measured by real-time RT-PCR at 24 h post-infection. (B) Kinetics of DENV RNA expression were determined by real-time RT-PCR. Relative expression of DENV RNA in AP-1A knockdown cells was compared with control cells. (C) Knockdown efficiency of AP-1A siRNA was examined by real-time RT-PCR. The results were plotted relative to cells transfected with control siRNA. Statistical significance was analyzed using the unpaired *t* test (*P<0.05). Error bars represent SEM from three independent experiments.

We compared viral RNA in Huh7 cells transfected with AP-1A siRNA or control siRNA at 24 h post-infection. Viral RNA level in Huh7 cells transfected with AP-1A siRNA was reduced by ~80% compared with that in cells transfected with control siRNA (Fig 4A), implying a role of AP-1A in DENV replication. We next determined how early AP-1A became involved in DENV replication. Huh7 cells transfected with AP-1A siRNA or control siRNA were infected with DENV-2 at a MOI of 1 for 2 h. Cells were harvested to measure DENV RNA by real-time RT-PCR at 0, 4, 8 and 12 h. Relative to the Huh7 cells transfected with control siRNA, DENV RNA level was lower in cells transfected with AP-1A siRNA at 8 and 12 h post-infection (Fig 4B), suggesting early involvement of AP-1A in DENV replication. Expression of AP-1A post-infection is shown in Fig 4C, which confirms that AP-1A was knocked down at each time point.

**Fig 5:**
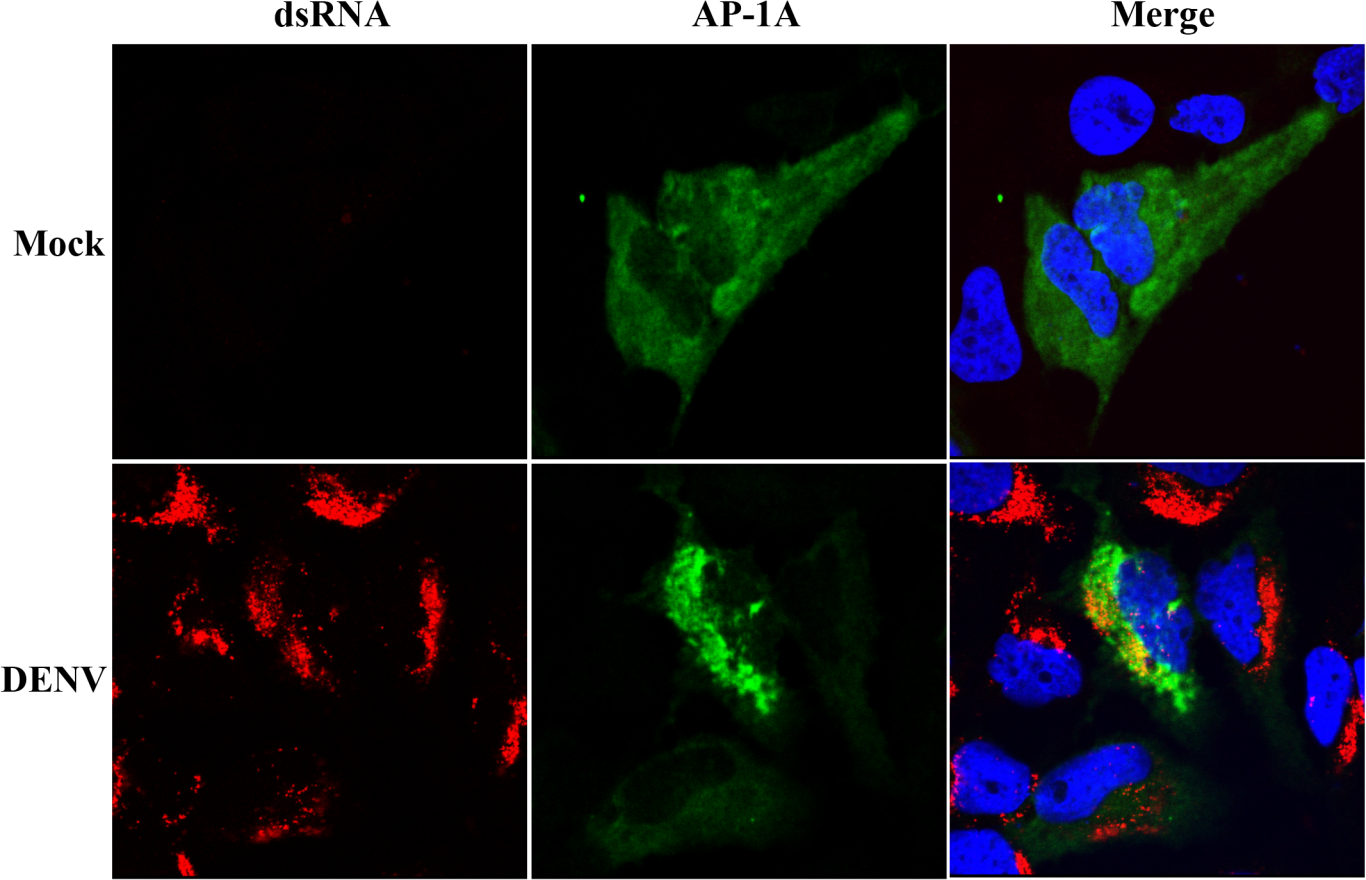
AP-1A was partially co-localized with dsRNA in DENV-infected cells. Huh7 cells were plated on coverslips, transfected with a plasmid containing AP-1A [47] and infected with DENV for 24 h. The cells were fixed and incubated with anti-dsRNA antibody and anti-GFP antibody, Upon removal of primary antibodies, cells were incubated with Alexa Fluor 488-conjugated donkey anti-rabbit IgG and Alexa Fluor 594-conjugated donkey anti-mouse IgG Hoechst 33342 was used to stain nuclei of the cells. The cells were visualized by a confocal laser-scanning microscope (LSM 510 Meta).

Immunofluoresence assay was subsequently performed to determine whether AP-1A is co-localizaed with viral RNA at the replication site during DENV infection. The result shows that AP-1A was partially co-localized with dsRNA near the perinuclear region of DENV-infected Huh7 cells (Fig 5) suggesting that AP-1A may be recruited into the replication site during DENV infection.

**Fig 6:**
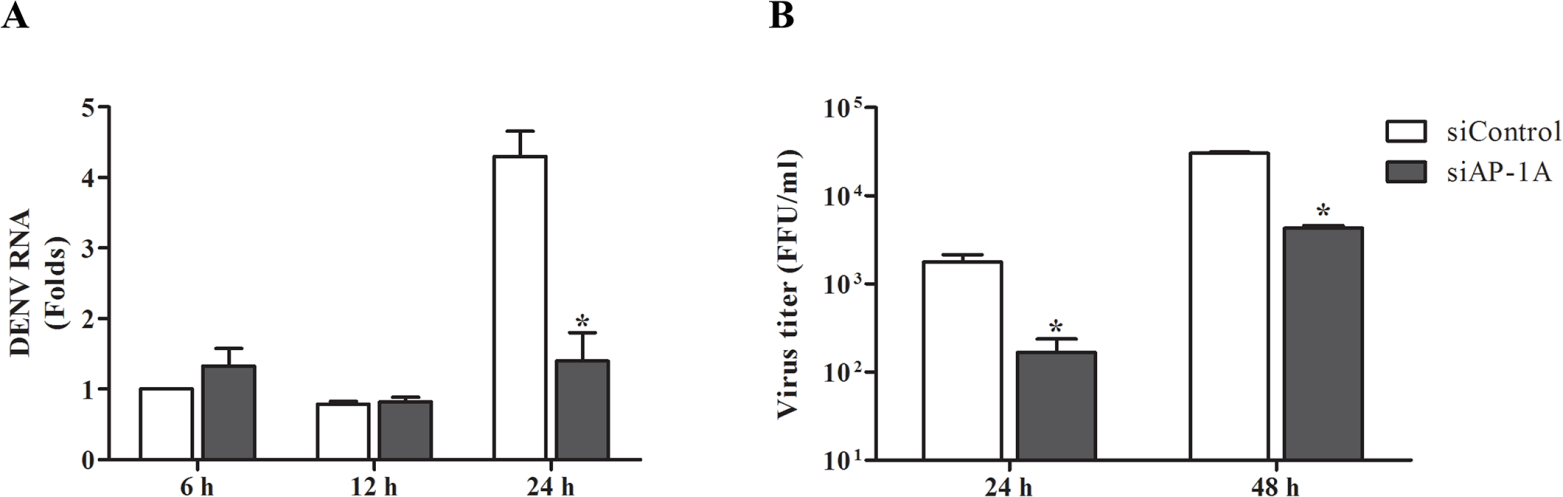
Depletion of AP-1A impaired DENV genome replication. (A) Effect of AP-1A siRNA on DENV RNA synthesis. Cells transfected with control and AP-1A siRNA were transfected with 0.5 μg DENV RNA using Lipofectamine 2000. Viral RNA level was determined by real-time RT-PCR at 6, 12 and 24 h post-transfection. (B) Quantification of virions released from cells transfected with DENV RNA. At 24 h and 48 h post-transfection, culture supernatants were collected for titration by FFU assay. The results were plotted relative to cells transfected with control siRNA. Statistical significance was analyzed using unpaired *t* test (*P<0.05). Error bars represent SEM from three independent experiments.

Naked DENV RNA was directly transfected into Huh7 cells transfected with AP-1A siRNA or control siRNA to exclude the role of AP-1A in viral fusion and uncoating. Viral RNA was measured by real-time RT-PCR at 6, 12 and 24 h post-transfection. DENV RNA was significantly decreased in Huh7 cells transfected with AP-1A siRNA compared with control siRNA at 24 h post-transfection, suggesting that naked DENV RNA replicates in Huh7 cells, but DENV RNA replication was decreased in the absence of AP-1A (Fig 6A). FFU assay was subsequently performed. Naked DENV RNA produced infectious virions in Huh7 cells transfected with control siRNA, but production was significantly decreased in the absence of AP-1A (Fig 6B).

**Fig 7:**
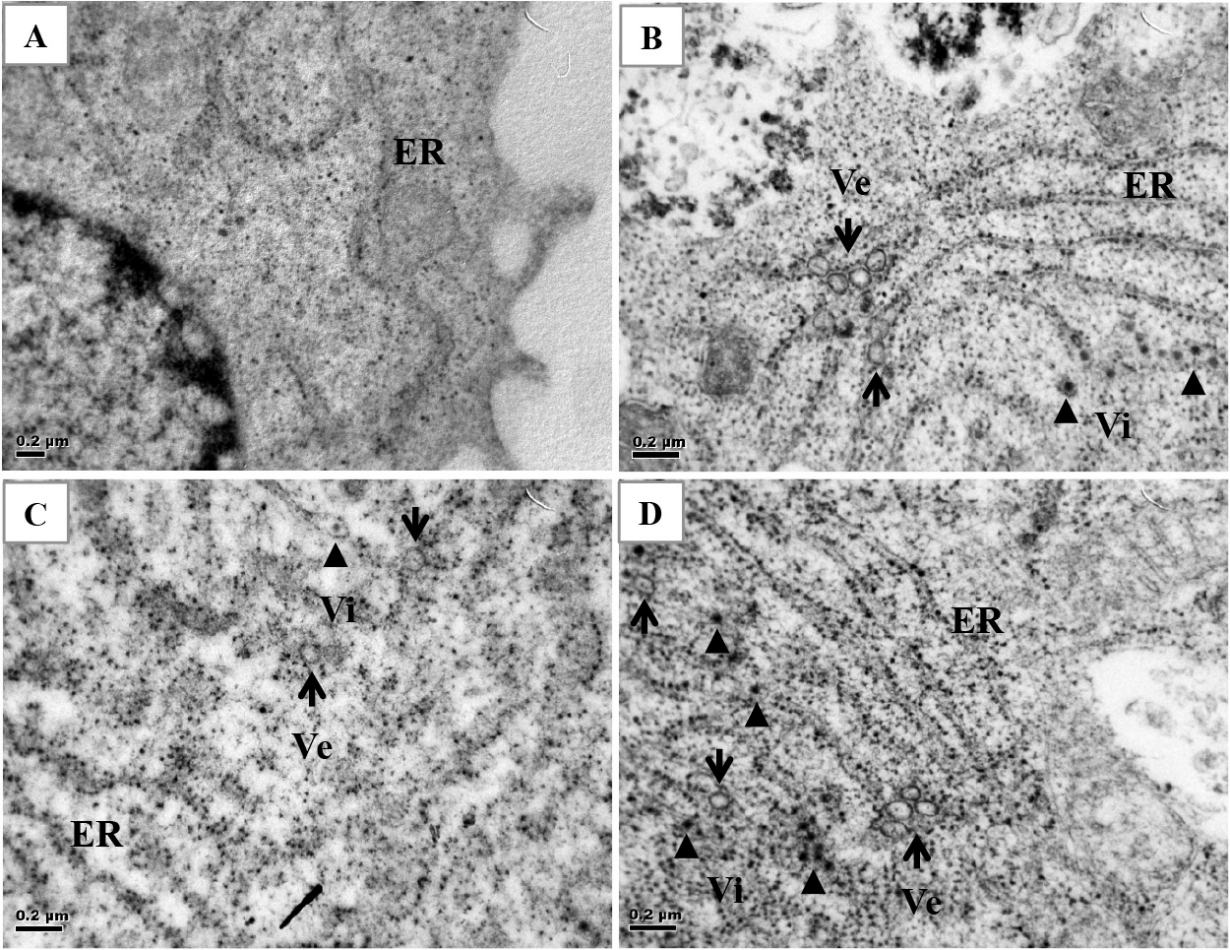
AP-1A knockdown affected the DENV replication site. (A) Ultrastructural analysis of Huh7 cells transfected with control siRNA was observed by TEM at 48 h after second transfection. (B) Cells transfected with control siRNA. (C) Cells transfected with AP-1A siRNA. (D) Cells transfected with AP-2 siRNA were infected with DENV-2 at a MOI of 10 for 24 h. Cells were fixed, processed and analyzed by TEM. Ve, virus-induced vesicles (arrow); Vi, virus particles (arrowhead).

TEM was performed to determine the morphology of Huh7 cells transfected with AP-1A siRNA or control siRNA and infected with DENV at a MOI of 10. Compared with mock-infected cells (Fig 7A), DENV-infected Huh7 cells at 24 h post-infection had virus particles, and modification of ER membranes including vesicular packets (Fig 7B). However, these packets were reduced in number in Huh7 cells transfected with AP-1A siRNA (Fig 7C). Modification of ER membranes was conserved in Huh7 cells transfected with AP-2 siRNA (Fig 7D). Our data indicated the role of AP-1A in replication of DENV.

**Fig 8:**
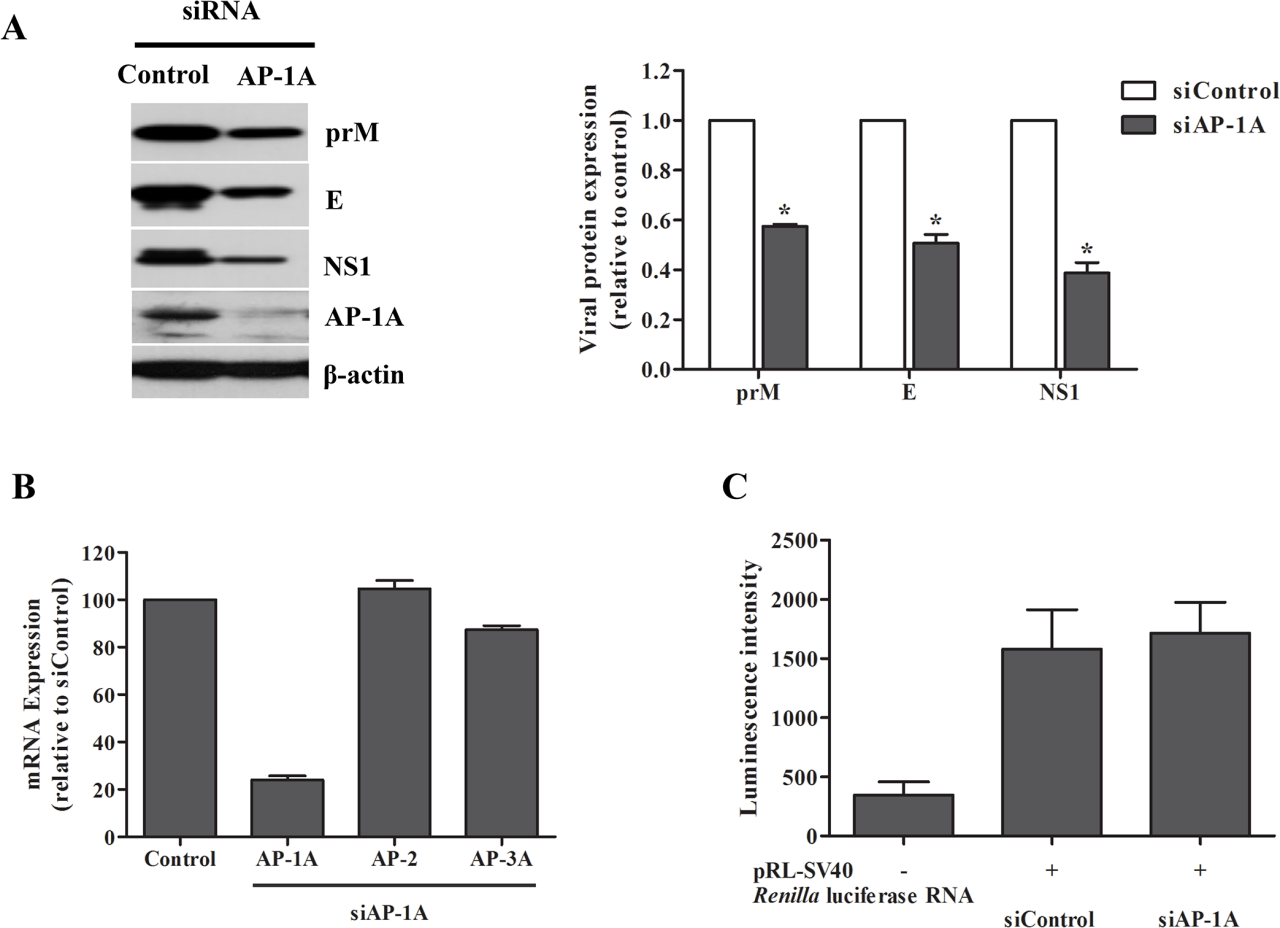
Expression of DENV protein was decreased in Huh7 cells transfected with AP-1A siRNA. (A) Huh7 cells were transfected with control siRNA and AP-1A siRNA and infected with DENV-2 for 24 h. DENV proteins were examined at 24 h post-infection by western blotting. Band intensity of DENV proteins was quantified using Image J software. (B) Expression of AP-1A, AP-2 or AP-3A in Huh7 cells was examined by real-time RT-PCR at 48 h after second transfection. (C) pRL-SV40 vector, which contains *Renilla* luciferase gene, was subjected to *in vitro* transcription. To determine the effect of AP-1A knockdown on translation, Huh7 cells were transfected twice with AP-1A-specific siRNA or control siRNA. After the second round of siRNA transfection, cells were transfected with 2.5 nM reporter RNA followed by replacement with fresh culture medium at 4 h later. Following 8 h after transfection with reporter RNA, cells were harvested and determined for *Renilla* luciferase expression using Luciferase Reporter Assay System (Promega).

We verified whether DENV protein expression was affected after DENV RNA replication. Western blotting was performed using lysates prepared from DENV-infected or mock-infected Huh7 cells in the presence or absence of AP-1A. Expression of DENV prM, DENV E and DENV NS1 in Huh7 cells transfected with AP-1A siRNA was decreased compared with that in cells transfected with control siRNA (Fig 8A). We further tested whether host transcription and translation were compromised by AP-1A disruption by real-time RT-PCR using primers specific to AP-2, and AP-3A and by translation assay, respectively. The result in Fig 8B demonstrated that the mRNA expression of AP-2, and AP-3A was not compromised by AP-1A disruption. Furthermore, host translation machinery was not compromised by AP-1A disruption as luminescence intensity of *Renilla* luciferase activities between Huh7 cells transfected with AP-1A siRNA or with control siRNA were relatively similar (Fig 8C). As ER stress may be activated during AP-1A disruption thereby leading to translation inhibition, which could reduce expression of viral proteins, western blot analysis was performed using lysates form DENV-infected Huh7 cells in the presence or absence of AP-1-dependent traffic inhibitor (A5), the result shows that GRP78 protein expression was relatively similar (S1 Fig); therefore, AP-1A disruption may not lead to translation inhibition to reduce viral protein expression. All data suggest that DENV protein expression was reduced after DENV RNA replication in Huh7 cells transfected with AP-1A siRNA.

**Fig 9:**
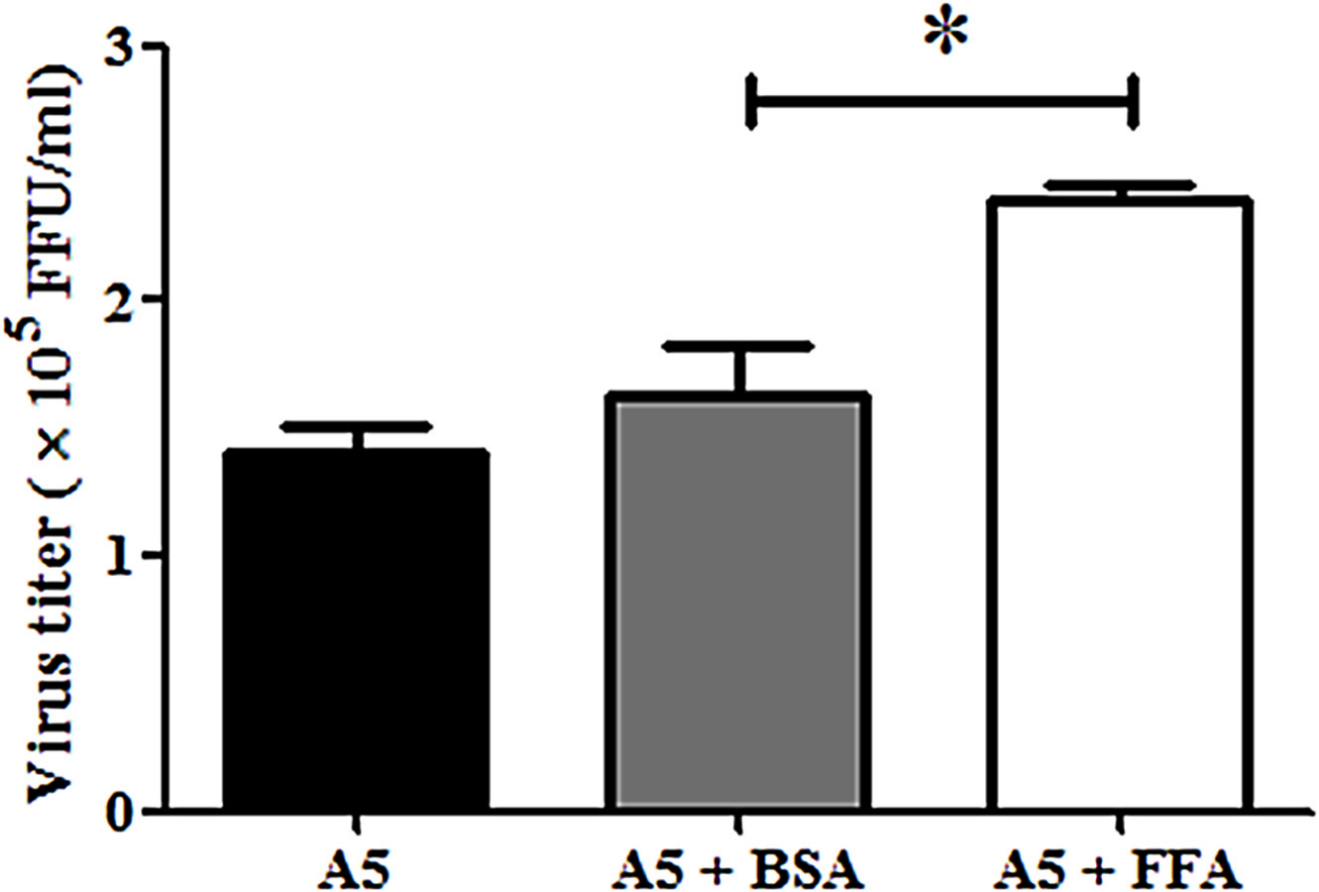
Exogenous fatty acid increased DENV production after A5 treatment. DENV-infected Huh7 cells were incubated with A5 in the presence of oleic acid-BSA or fatty acid free-BSA for 24 h. The culture supernatants were collected for FFU assay. Statistical significance was analyzed using the unpaired *t* test. *P<0.05. Error bars represent SEM from three independent experiments.

Enhanced fatty acid synthesis is required for efficient membrane proliferation and rearrangement. Rearrangement of membrane structure induced by dengue virus (DENV) is essential for replication, and requires host cellular machinery [25, 48, 49]. We next asked whether disturbance of AP-1 by A5 affect fatty acid synthesis, which is essential for dengue viral replication. Lipid complementation assay was performed. DENV-infected Huh7 cells were incubated with A5 in the presence of oleic acid-BSA or fatty acid free-BSA for 24 h. The culture supernatants were collected for FFU assay. Oleic acid-BSA could increase DENV production compared to fatty acid free-BSA (Fig 9) suggesting that AP-1 may involve in lipid synthesis required for DENV replication.

**Fig 10:**
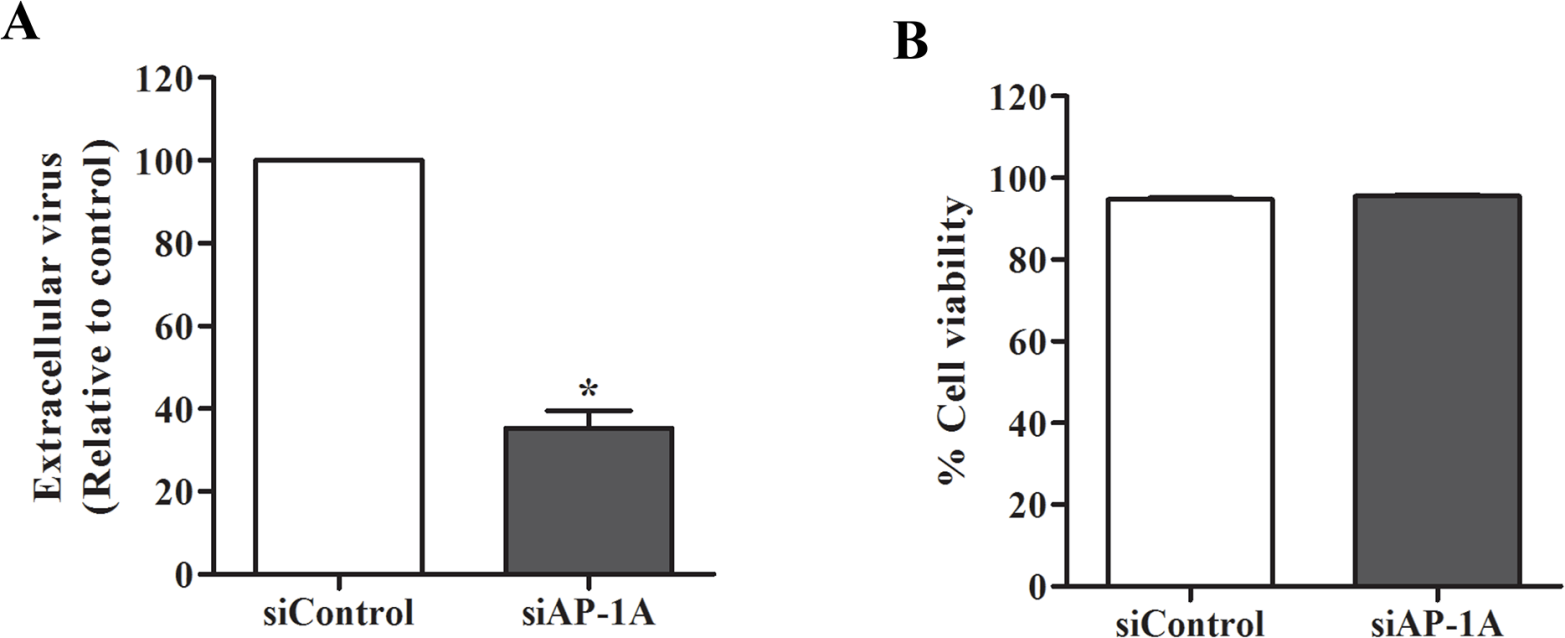
Silencing of AP-1A reduced virus production. Huh7 cells were transfected with control siRNA and AP-1A siRNA and infected with DENV-2 for 24 h. (A) Virus titer in culture supernatants was measured by FFU assay. (B) Cell viability was measured by trypan blue exclusion. Statistical significance was analyzed using unpaired *t* test. *P<0.05. Error bars represent SEM from three independent experiments.

The final step was to verify DENV production in Huh7 cells transfected with AP-1A siRNA compared with control siRNA. Although the number of viable cells was similar, AP-1A siRNA decreased the yield of viral progeny compared with that of control siRNA (Fig 10A and 10B).

### AP-1 is involved in virion production for all serotypes

**Fig 11:**
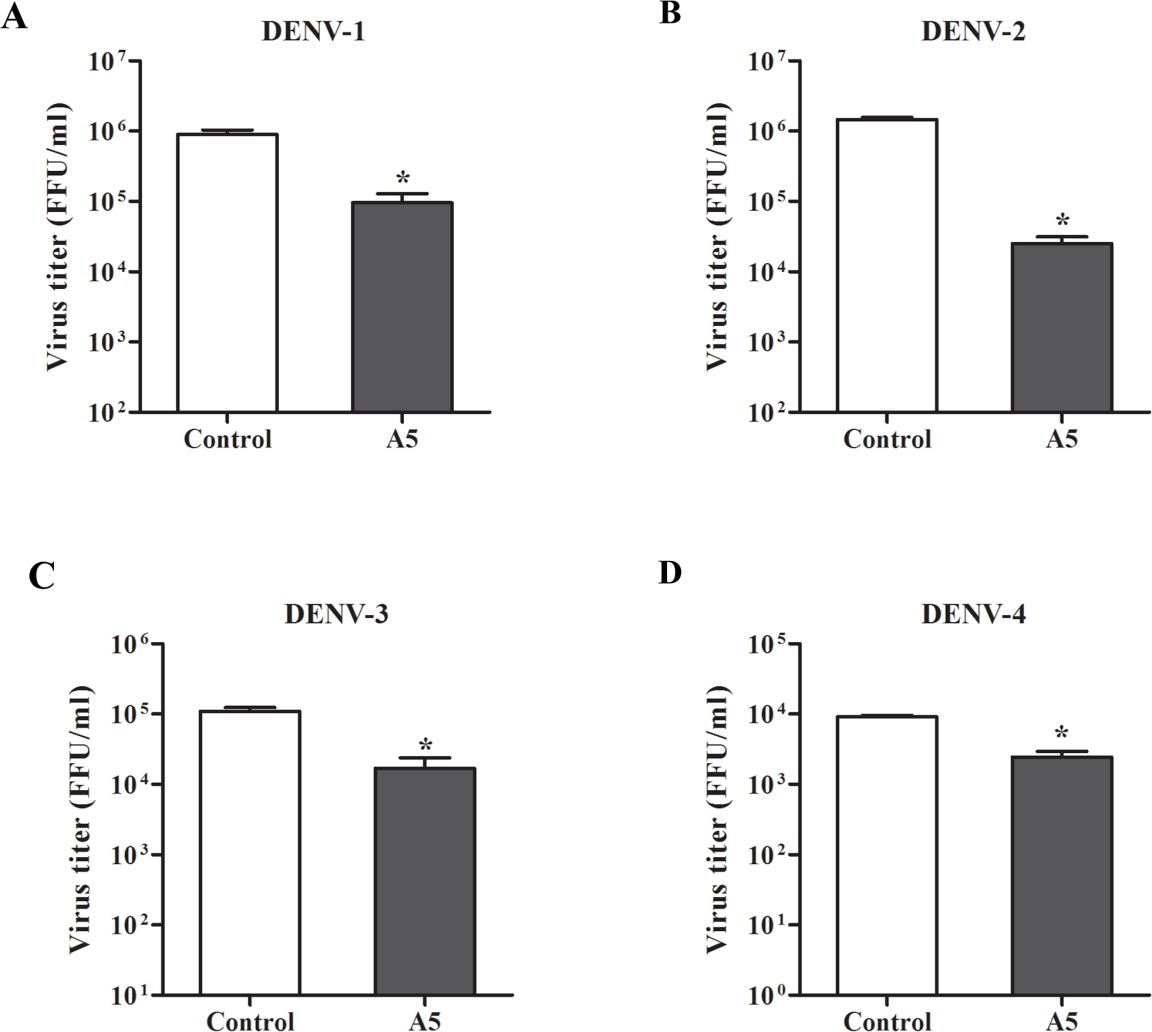
AP-1 was involved in production of four serotypes of DENV. Inhibitory effect of A5 on virus replication was determined in all four serotypes of DENV. Huh7 cells were infected with DENV-1, -2, -3 and -4 at a MOI of 1 for 2 h. Unbound virus was removed by washing with PBS. DENV-infected cells were incubated with A5 (200 μM) or culture medium (control) for 24 h. Virus titer in culture supernatants was measured by FFU assay. (A) Titer of DENV-1; (B) titer of DENV-2; (C) titer of DENV-3; (D) titer of DENV-4. Statistical significance was analyzed using unpaired *t* test (*P<0.05). Error bars represent SEM from three independent experiments.

To determine whether AP-1-dependent traffic plays a role in four serotypes of DENV, Huh7 cells were infected with each DENV serotype, followed by treatment with AP-1-dependent traffic inhibitor (A5).The titer of DENV was measured in culture supernatant by FFU assay. A5 had an inhibitory effect on all serotypes of DENV (Fig 11A–11D). However, the reduction for DENV-2 was greater than for the other serotypes.

## Discussion

Using a human-genome-wide RNAi screen, clathrin and its adaptor proteins were shown to decrease DENV infection [22]. A pathway-specific siRNA library further revealed the role of clathrin and its adaptor proteins in mediating DENV entry [4] and secretion of subviral particles [24]. Furthermore, the role of AP-1A in DENV production was shown to play a role at the egress stage from the TGN to plasma membrane [38]. In the present study, we showed that treatment with AP-1-dependent traffic inhibitor (A5), or transfection with AP-1A siRNA decreased replication of DENV, thereby reducing viral protein expression and production. Thus, AP-1 may have an additional role besides aiding egression of DENV, as shown previously [38]. This hypothesis was supported by RNAi, which showed that DENV RNA was significantly reduced in DENV-infected Huh7 cells transfected with AP-1A siRNA compared with control siRNA. Naked DENV RNA transfection, which bypassed the process of viral fusion and uncoating, demonstrated decreased production of viral RNA and infectious virions in cells transfected with AP-1A siRNA compared with control siRNA-transfected cells. This was indicative of an essential function of AP-1A in the step of DENV RNA replication. Vesicular packets, which are a proposed replication site for DENV, were fewer in number in Huh7 cells transfected with AP-1A siRNA compared with control siRNA.

AP-1, GTPase ADP-ribosylation factor 1 (ARF)-1 and phosphatidylinositol-4-phosphate (PI4P) are the components, which are essential for reorganization of donor membrane for clathrin-coated vesicle [50]. AP-1A and AP-3A are required for transport between endosomal/lysosomal systems and the secretory pathway [51, 52]. AP-3A was previously shown to be involved in replication of DENV [38], therefore, we proposed here that AP-1A may act in concert with AP-3A to facilitate replication of DENV. AP-1A and AP-3A coat assembly are controlled by GTPase ARF-1 [53, 54]. ARF-1 plays a key role in trafficking through the Golgi apparatus, where it is involved in the formation of vesicular packets, and ARF family siRNAs have an inhibitory effect on DENV recombinant subviral particle secretion [24, 55]. Rab18, a GTPase involved in vesicular trafficking, also regulates DENV replication by targeting enzymes required for cellular fatty acid synthesis to the replication site [56]. Enhanced fatty acid synthesis is required for efficient membrane proliferation and rearrangement in DENV replication [25, 26, 57]. Recruitment of PI4P is also required for membrane reorganization [58]. DENV may use AP-1A to recruit enzymes (PI4K-IIIβ) for synthesis of PI4P to help its own replication. Purified AP-1 binds to PI4P, and anti-PI4P inhibits recruitment of cytosolic AP-1 to normal cellular membranes [59]; therefore, disruption of AP-1A by RNAi in the present study may have affected synthesis of PI4P and membrane organization required for DENV replication. The role of phosphatidylinositol-4-kinases, including PI4K-IIIα, as a modulator of hepatitis C virus (HCV), was demonstrated by co-localization of PI4K-IIIα and HCV NS5A in lipid rafts. Inhibition of web formation by siRNA against PI4K-IIIα correlates with the decrease in HCV replication and infectious virion production. PI4K-IIIα is proposed to produce pools of PI4P for HCV replication [60]. In addition, DENV can activate autophagic machinery for viral replication both *in vitro* and *in vivo* [61, 62]. DENV infection can induce an autophagy-dependent processing of lipid droplets and triglycerides to release free fatty acids for replication [26], linking of DENV replication through autophagolysosome was demonstrated [63], and dysfunction of the AP-1A-dependent clathrin coating at the TGN can prevent autophagosome formation [64]. AP-1A may be a host component, which can recruit enzymes required for fatty acid synthesis and dysfunction of AP-1A may affect membrane organization, thereby decreasing replication of virus in infected cells.

## Conclusion

AP-1A was characterized to establish its role during the DENV life cycle, using an inhibitor and RNAi. RNAi specific to AP-1A decreased viral RNA and protein levels, and virion production in Huh7 cells.

## Supporting Information

S1 Fig AP-1-dependent traffic inhibitor, A5, did not induce ER stress. Huh7 cells were infected with DENV-2 at a MOI of 1 for 2 h. Unbound virus was removed by washing with PBS. Mock- or DENV-infected Huh7 cells were incubated with A5 at different concentrations (0, 100 or 200 μM) for 48 h. The cells were lysed and subjected to western blot analysis using antibodies specific to human GRP78 and β-actin.
(TIF)

S1 Table Primers used for real-time RT-PCR analysis. RNA was extracted from DENV-infected Huh7 cells, which were transfected with AP-1A-specific siRNA or control siRNA. After cDNA synthesis, Real-time RT-PCR was performed using primers listed in S1 Table. Relative levels of human AP-1A, AP-2, AP-3A mRNA and viral RNA expression were determined by normalization to the expression levels of human β-actin.
(PDF)
