# Supplementary figures and images for: Adaptor Protein 1A Facilitates Dengue Virus Replication
Source: PLoS One. 2015 Jun 19;10(6):e0130065. doi: 10.1371/journal.pone.0130065 (PMC4474434; doi:10.1371/journal.pone.0130065)

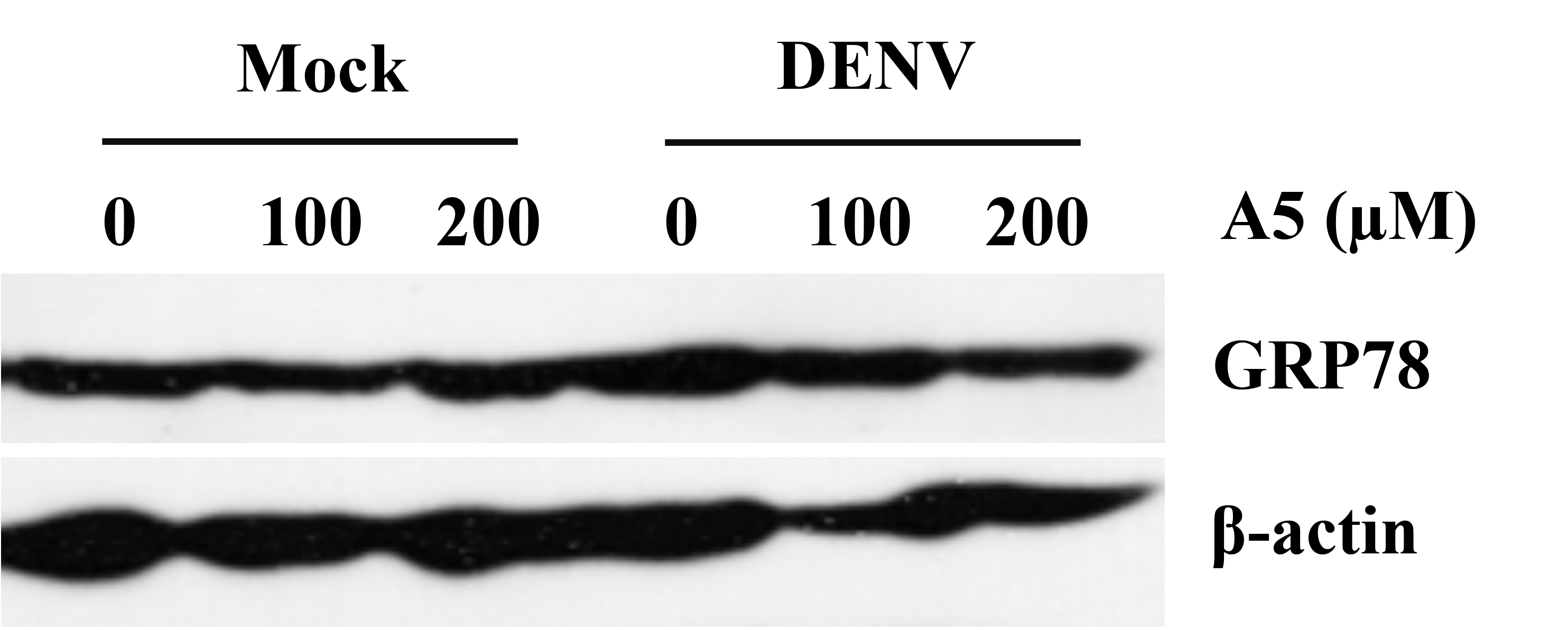

Supplement: S1 Fig — Huh7 cells were infected with DENV-2 at a MOI of 1 for 2 h. Unbound virus was removed by washing with PBS. Mock- or DENV-infected Huh7 cells were incubated with A5 at different concentrations (0, 100 or 200 μM) for 48 h. The cells were lysed and subjected to western blot analysis using antibodies specific to human GRP78 and β-actin. (TIF) [file pone.0130065.s001.tif]
