# Supplementary material for: Adaptor Protein 1A Facilitates Dengue Virus Replication
Source: PLoS One. 2015 Jun 19;10(6):e0130065. doi: 10.1371/journal.pone.0130065 (PMC4474434; doi:10.1371/journal.pone.0130065)
Supplement: S1 Table — RNA was extracted from DENV-infected Huh7 cells, which were transfected with AP-1A-specific siRNA or control siRNA. After cDNA synthesis, Real-time RT-PCR was performed using primers listed in S1 Table. Relative levels of human AP-1A, AP-2, AP-3A mRNA and viral RNA expression were determined by normalization to the expression levels of human β-actin. (PDF) [file pone.0130065.s002.pdf]

Table 1 Oligonucleotide primers

Genes	Forward primers	Reverse primers
AP-1A	5’-CTAGTGTGGAGGCCGAAGAC-3’	5’-CGGAGCTGGTAATCTCCATT-3’
AP-2	5’-CAGCAGGGCATCAAGAGTCAGCA-3’	5’-CACCTGCCCTTGTGGGGACATGA-3’
AP-3A	5’-ATGAACCTGACACCCACACA-3’	5’-GGCTCGGATTCTCTTCTGGT-3’
DENV E	5’-ATCCAGATGTCATCAGGAAAC-3’	5’-CCGGCTCTACTCCTATGATG-3’
ACTB	5’-AGAAAATCTGGCACCACACC-3’	5’-CTCCTTAATGTCACGCACGA-3’
